# Glucocorticoid activation by 11β‐hydroxysteroid dehydrogenase enzymes in relation to inflammation and glycaemic control in chronic kidney disease: A cross‐sectional study

**DOI:** 10.1111/cen.13889

**Published:** 2018-11-15

**Authors:** Michael S. Sagmeister, Angela E. Taylor, Anthony Fenton, Nadezhda A. Wall, Dimitrios Chanouzas, Peter G. Nightingale, Charles J. Ferro, Wiebke Arlt, Paul Cockwell, Rowan S. Hardy, Lorraine Harper

**Affiliations:** ^1^ Institute of Inflammation and Ageing University of Birmingham Birmingham UK; ^2^ Department of Renal Medicine University Hospitals Birmingham NHS Foundation Trust Birmingham UK; ^3^ Institute of Metabolism and Systems Research University of Birmingham Birmingham UK; ^4^ Institute of Clinical Sciences University of Birmingham Birmingham UK; ^5^ Institute of Translational Medicine University Hospitals Birmingham NHS Foundation Trust Birmingham UK; ^6^ Institute of Inflammation and Ageing, ARUK Rheumatoid Arthritis Centre of Excellence, and MRC ARUK Centre for Musculoskeletal Ageing University of Birmingham Birmingham UK

## Abstract

**Objective:**

Patients with chronic kidney disease (CKD) have dysregulated cortisol metabolism secondary to changes in 11β‐hydroxysteroid dehydrogenase (11β‐HSD) enzymes. The determinants of this and its clinical implications are poorly defined.

**Methods:**

We performed a cross‐sectional study to characterize shifts in cortisol metabolism in relation to renal function, inflammation and glycaemic control. Systemic activation of cortisol by 11β‐HSD was measured as the metabolite ratio (tetrahydrocortisol [THF]+5α‐tetrahydrocortisol [5αTHF])/tetrahydrocortisone (THE) in urine.

**Results:**

The cohort included 342 participants with a median age of 63 years, median estimated glomerular filtration rate (eGFR) of 28 mL/min/1.73 m^2^ and median urine albumin‐creatinine ratio of 35.5 mg/mmol. (THF+5αTHF)/THE correlated negatively with eGFR (Spearman's *ρ* = −0.116, *P* = 0.032) and positively with C‐reactive protein (*ρ* = 0.208, *P* < 0.001). In multivariable analysis, C‐reactive protein remained a significant independent predictor of (THF+5αTHF)/THE, but eGFR did not. Elevated (THF+5αTHF)/THE was associated with HbA1c (*ρ* = 0.144, *P* = 0.008) and diabetes mellitus (odds ratio for high vs low tertile of (THF+5αTHF)/THE 2.57, 95% confidence interval 1.47‐4.47). Associations with diabetes mellitus and with HbA1c among the diabetic subgroup were independent of eGFR, C‐reactive protein, age, sex and ethnicity.

**Conclusions:**

In summary, glucocorticoid activation by 11β‐HSD in our cohort comprising a spectrum of renal function was associated with inflammation and impaired glucose control.

## 1 INTRODUCTION

Glucocorticoids (GCs) are steroid hormones that play a critical role in regulating energy metabolism, inflammation, cardiovascular function and behavioural processes. In excess, GCs drive metabolic disease, insulin resistance and cardiovascular disease.^1^ Critical to the actions of GCs are their pre‐receptor metabolism by the 11beta‐hydroxysteroid dehydrogenase (11β‐HSD) type 1 and type 2 enzymes.^2^ 11β‐HSD1 is a bidirectional enzyme that primarily converts inactive GC (cortisone) to its active counterpart (cortisol) and is widely expressed throughout the body.^3^ In contrast, 11β‐HSD2 solely inactivates GCs and is primarily expressed in mineralocorticoid sensitive tissues such as the kidney. Together, these determine local tissue exposure.

Several studies have described a correlation between loss of renal function and GC activation by 11β‐HSD enzymes, measured as a rise in the ratio of cortisol/cortisone or a rise in the ratio of their respective metabolites (tetrahydrocortisol [THF]+5α‐tetrahydrocortisol [5αTHF])/tetrahydrocortisone (THE).^4^, ^5^ Due to limitations of measuring steroid metabolism in this way, the relative contribution of each 11β‐HSD enzyme to the reported shift in cortisol metabolism is not known. Loss of renal expression of 11β‐HSD2 and diminished cortisol inactivation likely plays a role.^6^ However, in vitro and animal models of uraemia propose concurrent 11β‐HSD1 upregulation and associated cortisol generation.^7^, ^8^

Chronic kidney disease (CKD) entails a pro‐inflammatory state.^9^ Pro‐inflammatory cytokines are potent inducers of 11β‐HSD1 activity, promoting local GC activation both in vitro and in vivo.^10^, ^11^ Upregulation of 11β‐HSD1 expression occurs in several human inflammatory conditions with a strong correlation between systemic measures of inflammation and increases in cortisol activation.^12^, ^14^ Existing studies in CKD have not evaluated inflammation as a driver of 11β‐HSD1 activity or GC activation.

The inflammatory activation in CKD contributes to insulin resistance.^15^, ^16^ Insulin resistance manifests from the early stages of renal impairment, and diabetes mellitus co‐exists in one third of people with CKD.^16^, ^18^, ^19^ Disturbances in glucose metabolism in the context of CKD are independently associated with cardiovascular disease, accelerated loss of renal function and death.^16^, ^18^, ^19^ Elevated 11β‐HSD1 activity has been implicated in metabolic disease and insulin resistance.^23^, ^24^ Suppression of 11β‐HSD1 activity protects against insulin resistance in murine models, including models of uraemia, and has improved HbA1c in Phase II clinical trials for type 2 diabetes.^7^, ^26^, ^27^ Whether elevated 11β‐HSD1 activity is involved in insulin resistance in the setting of human CKD has not yet been explored.

Given the central role of inflammation in regulating cortisol activation via 11β‐HSD1, we investigated its association with shifts in the cortisol‐cortisone equilibrium in human CKD. Furthermore, we examined whether augmented GC activation in CKD relates to impaired glucose handling. To test this, we performed a cross‐sectional study with 342 participants representing a broad spectrum of renal function. Using urine (THF+5αTHF)/THE as a validated indicator of systemic 11β‐HSD‐mediated GC activation, we examined its correlation with renal function, inflammation and glycaemic control in CKD.^5^, ^30^, ^31^

## 2 MATERIALS AND METHODS

### 2.1 Participants

This study included 331 participants with CKD and 11 healthy individuals without kidney disease. The Renal Impairment in Secondary Care (RIISC) observational study cohort provided the sampling frame for cases with CKD. Patient enrolment for RIISC took place at two teaching hospitals in Birmingham, United Kingdom, between 2010 and 2015. The eligibility criteria have been described in detail elsewhere.^33^ In brief, patients in RIISC had CKD stage 3 with declining estimated glomerular filtration rate (eGFR) of ≥5 mL/min/1.73 m^2^/y or ≥10 mL/min/1.73 m^2^/5 y or proteinuria with urinary albumin‐creatinine ratio (ACR) ≥70 mg/mmol, or CKD stage 4/5. Patients receiving renal replacement therapy or immunosuppressive treatment were excluded from the RIISC cohort. From a total of 932 cases in the RIISC cohort, 299 cases with incomplete biochemical data sets and 25 cases receiving systemic GC treatment were excluded prior to sampling. In total, 333 cases of 608 eligible cases were sampled for this study to obtain the desired sample size (see Statistical Analysis for power calculation). A stratified random sampling procedure was employed to balance the proportions of cases with eGFR ≤15, 15‐30, 30‐45 and ≥45 mL/min/m^2^ and to avoid oversampling cases from a narrow band of low eGFR which were preferentially enrolled in the RIISC cohort. Two cases had to be excluded in the experimental process due to technical failures.

Healthy individuals were recruited from the control arm of an ongoing study at the University of Birmingham characterizing immune dysfunction in older patients with CKD. These individuals did not have any known renal or inflammatory disease and were not using systemic GC treatment at the time of study enrolment. The rationale for inclusion of a small group of healthy individuals in analyses was to gain better insight into how changes in renal function affect glucocorticoid activation by 11β‐HSD, as cases with normal renal function and no active inflammation are underrepresented in the RIISC cohort.

Use of data and samples for this research falls under ethical approvals granted by the South Birmingham Local Research Ethics committee (participants with CKD; reference 10/H1207/6) and the Edgbaston Research Ethics Committee (participants without CKD; reference 15/WM/0057). All participants provided written informed consent, and the study was conducted in accordance with the Declaration of Helsinki.

### 2.2 Baseline assessment

Study participants' demographic information, past medical history and concomitant medications were taken from medical records. Diabetes mellitus was defined by history of diet‐controlled diabetes, treatment with anti‐diabetic medications or HbA1c greater than 48 mmol/mol.

Laboratory measurements included routine blood haematological profiles (Haematology Analyser, Beckman Coulter, Brea, CA, USA) and blood biochemical profiles and urinary ACR (Roche Hitachi 702 Analyser, Basel, Switzerland). eGFR was derived from the serum creatinine‐based CKD‐EPI equation. CRP was measured using the Full Range C‐Reactive Protein SpaPlus assay (The Binding Site, Birmingham, UK) for participants with CKD and the CRP High Sensitive ELISA (IBL, Hamburg, Germany) for volunteers without renal disease.

### 2.3 Urinary steroid measurements

The balance of systemic (ie, total body) cortisol‐cortisone interconversion by 11β‐HSD enzymes was taken as the ratio calculated from the urinary excretion of (THF+5αTHF)/THE. This methodology is well established as reflective of 11β‐HSD1 activity and has previously been validated in the literature,^30^, ^31^ including the use of early morning spot urine samples.^5^, ^34^

Spot urine samples were collected at morning clinic visits and stored at −80°C until analysis. Steroids were extracted from 400 µL of urine based on a previously reported protocol.^35^ The urine was spiked with 0.4 µg of internal standard (THE‐d5, THF‐d5; purchased from Isosciences, Ambler, PA, USA), and the steroids hydrolysed to remove their conjugate groups (sulphate and/or glucuronide). This was achieved by heating the urine for 3 hours at 60°C in a hydrolysis buffer (400 μL of 0.2 mol/L acetate buffer pH 4.8‐5: with 10 mg sulphatase [adjusted based on batch activity] and 10 mg ascorbate/3 mL of solvent). The now “free” deconjugated steroids were subsequently extracted by solid phase extraction using 96‐well C_18_ cartridges in a positive pressure manifold, eluting with 1 mL of methanol. The samples were dried at 50°C under nitrogen and reconstituted in 125 µL of 50/50 methanol/water for analysis with liquid chromatography‐tandem mass spectrometry (uPLC‐MS/MS).

A Waters Xevo mass spectrometer coupled to an Acquity uPLC with an electrospray ionization source in positive ionization mode was utilized in these experiments. THE, THF and 5αTHF were separated on a BEH C18 1.7 µm 5 cm column at 60°C with the mobile phases methanol and water both with 0.1% formic acid. Gradient profile was starting at 30% methanol, hold for one minute then a linear gradient to 60% methanol at 5 minutes, followed by washing and re‐equilibration steps. Steroids were identified by comparison to authentic reference standards, (THE, THF and 5αTHF purchased from Steraloids, Newport, RO, USA), with a matching retention time and identical mass transitions (MRMs) required for positive identification (Supporting Information Table S1 and Figure S1). Steroids were quantified relative to a calibration series ranging from 10‐5000 ng/mL prepared in synthetic urine, including a blank. Steroid concentrations were calculated relative to an assigned internal standard. Validation of this method is described in the supplementary section (Supporting Information Table S2). Measurements of urinary cortisol and cortisone for supplementary analysis followed similar principles, and full details for this methodology are provided in the supplementary materials (Supporting Information Appendix S1, Table S1 and Figure S2).

### 2.4 Statistical analysis

Data for continuous variables were not normally distributed. Mann‐Whitney *U* test, Kruskal‐Wallis test followed by Dunn's multiple comparisons test and Spearman's correlation test were used as appropriate. For multiple linear regression analysis, outcome variables were log‐transformed to comply with the requisite statistical assumptions. For binary logistic regression analysis, urinary (THF+5αTHF)/THE ratio, eGFR and CRP did not comply with the linearity assumption of independent variables and log odds. These were therefore included as log‐transformed variables (CRP) or as stratified variables (tertiles for urine (THF+5αTHF)/THE with limits ≤0.983, 0.983‐1.586 and >1.586; clinical CKD stages for eGFR with ≤15, 15‐30, 30‐45 and >45 mL/min/1.73 m^2^). Cases with missing values were not included in multivariable analyses, but this applied to ≤3.2% of cases for any reported model. Results were deemed statistically significant at *P* < 0.05. Sample size estimation considered that a covariate added to a baseline linear regression model for urine (THF+5αTHF)/THE with assumed *R* ^2^ = 0.10 producing an increment to *R* ^2^ ≥ 0.025 will be identified as statistically significant at *P* ≤ 0.05 with power 0.80 for n = 282. The final sample size included a redundancy margin of 15‐20 per cent. Analyses were performed using IBM SPSS Statistics for Windows Version 23 (IBM Corp; Armonk, NY, USA).

## 3 RESULTS

The clinical and laboratory characteristics of the study participants are summarized in Table 1 (for break‐down by aetiology of renal disease see Supporting Information Table S3).

**Table 1:** Cohort characteristics

Variable	Value
n	342
Age (y)	63 (50‐75)
Female	134 (39%)
Ethnicity	
White	242 (71%)
South Asian	62 (18%)
Black	35 (10%)
Other	3 (1%)
Aetiology of renal disease	
Ischaemic/hypertensive	70 (20%)
Glomerulonephritis	70 (20%)
Polycystic	32 (9%)
Diabetic	31 (9%)
Obstructive/reflux	24 (7%)
Interstitial disease	19 (6%)
Not known and other	85 (25%)
No renal disease	11 (3%)
eGFR (mL/min/1.73 m^2^)	28 (18‐43)
Urine ACR (mg/mmol)	35.5 (5.4‐120.6)
Diabetes	122 (36%)
HbA1c (mmol/mol)	42 (38‐50)
C‐reactive protein (mcg/mL)	2.7 (1.2‐6.3)
Urine (THF+5αTHF)/THE	1.23 (0.86‐1.80)

Continuous variables are reported as median (interquartile range) and categorical variables as frequency (percentage).

ACR, albumin‐creatinine ratio; eGFR, estimated glomerular filtration rate; (THF+5αTHF)/THE, tetrahydrocortisol+5α‐tetrahydrocortisol/tetrahydrocortisone.

### 3.1 Renal diagnosis and function

**Figure 1:**
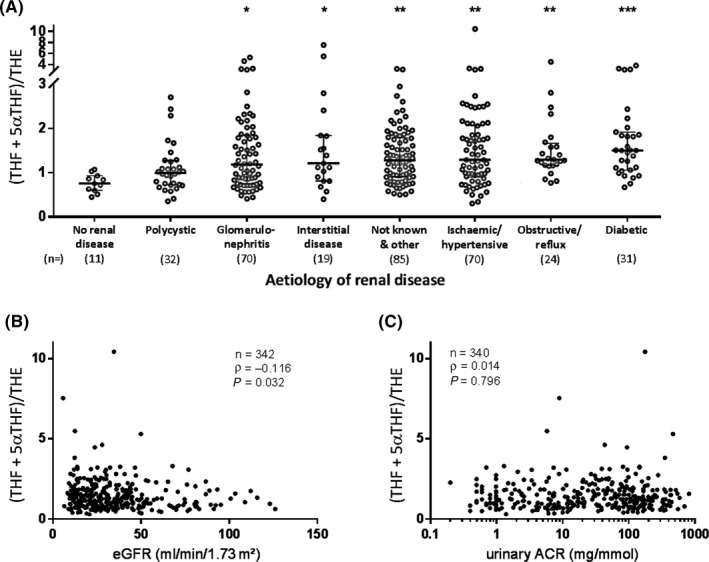
Association of urine (THF+5αTHF)/THE with renal diagnosis and function. A, Urine (THF+5αTHF)/THE is higher in renal disease regardless of aetiology, except for polycystic renal disease. Urine (THF+5αTHF)/THE is significantly different for polycystic vs diabetic renal disease, but not for other comparisons by aetiology of renal disease. (THF+5αTHF)/THE is displayed on a discontinuous scale to show outliers that were included in the analysis. Beams and whiskers indicate group medians and interquartile ranges, respectively; numbers in parentheses indicate cases per group. Group comparisons used Dunn's multiple comparisons test: *P < 0.05, **P < 0.01, ***P < 0.001 vs no renal disease. B, Urine (THF+5αTHF)/THE correlates negatively with renal function, but (C) does not correlate with albuminuria. Urinary ACR is displayed on a log scale. Spearman's test was used to assess correlations. ACR, albumin‐creatinine ratio; eGFR, estimated glomerular filtration rate; (THF+5αTHF)/THE, tetrahydrocortisol+5α‐tetrahydrocortisol/tetrahydrocortisone

Previous studies have reported increasing GC activation by 11β‐HSD enzymes with declining renal function,^4^, ^6^ which we tested in our larger cohort. Urine (THF+5αTHF)/THE was elevated in renal disease regardless of aetiology compared to healthy volunteers, except for polycystic renal disease (Figure 1A). Among cases with renal disease, urine (THF+5αTHF)/THE was lowest in polycystic renal disease and highest in diabetic nephropathy. As in previous reports, we found a negative but weak correlation of urine (THF+5αTHF)/THE with eGFR (*ρ *= −0.116, *P* = 0.032; Figure 1B). There was no association with urinary albumin‐creatinine ratio (ACR; *ρ* = 0.014, *P* = 0.796; Figure 1C). These results consolidate previous observations that people with renal impairment have higher GC activation by 11β‐HSD.

### 3.2 Inflammation

**Figure 2:**
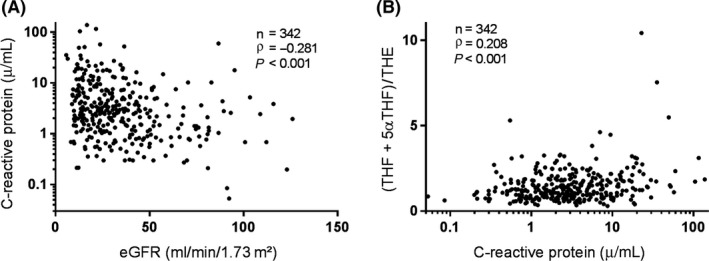
Correlation of inflammation with renal function and urine (THF+5αTHF)/THE. A, Inflammation, measured by C‐reactive protein, is negatively correlated with eGFR and (B) positively correlated with urine (THF+5αTHF)/THE ratio. C‐reactive protein is displayed on a log scale. Spearman's test was used to assess correlations. eGFR, estimated glomerular filtration rate; (THF+5αTHF)/THE, tetrahydrocortisol+5α‐tetrahydrocortisol/tetrahydrocortisone

Chronic kidney disease is known to entail a pro‐inflammatory state.^9^ This was apparent in our cohort in the negative correlation for eGFR with C‐reactive protein (CRP; *ρ* = −0.281, *P* < 0.001; Figure 2A). Importantly, we identified a significant positive correlation between CRP and urine (THF+5αTHF)/THE (*ρ* = 0.208, *P* < 0.001; Figure 2B). This is reflected in the earlier observation of relatively low urine (THF+5αTHF)/THE in patients with polycystic kidney disease, as this subgroup also exhibited low CRP (Supporting Information Table S3). We constructed a multivariable linear regression model to explore independent determinants of urine (THF+5αTHF)/THE. Covariates included demographic factors, markers of renal function (eGFR, urine ACR), inflammation (CRP) and glycaemic control (HbA1c) (Table 2). Inflammation remained independently associated with urine (THF+5αTHF)/THE. However, there was no independent relationship between eGFR and urine (THF+5αTHF)/THE in the multiple linear regression model. This suggests that GC activation by 11β‐HSD is more closely associated with inflammation than with renal function in this CKD cohort.

**Table 2:** Independent determinants of 11β‐HSD‐mediated glucocorticoid activation (n = 337)

Variable	*B*	SE	*β*	*t*	*P*
Age (y)	1.13 × 10^−3^	1.94 × 10^−3^	0.035	0.58	0.560
Sex					
Male	Reference				
Female	−0.143	0.058	−0.130	−2.47	0.014
Ethnicity					
White	Reference				
South Asian	0.104	0.078	0.074	1.33	0.185
Black	0.208	0.094	0.119	2.20	0.028
Other	−0.565	0.300	−0.099	−1.89	0.060
eGFR (mL/min/1.73 m^2^)	−1.76 × 10^−3^	1.34 × 10^−3^	−0.075	−1.31	0.191
Urine ACR (mg/mmol)	3.43 × 10^−5^	2.40 × 10^−4^	0.008	0.14	0.886
C‐reactive protein (mcg/ml)	6.72 × 10^−3^	2.14 × 10^−3^	0.172	3.14	0.002
HbA1c (mmol/mol)	3.08 × 10^−3^	2.39 × 10^−3^	0.074	1.29	0.198

Multivariable linear regression analysis with dependent variable log_e_‐transformed urine (THF+5αTHF)/THE. Adjusted *R* ^2 ^= 0.083. Exclusion of the 11 healthy volunteer cases from the analysis or substitution of HbA1c with diagnosis of diabetes does not significantly impact on the displayed parameters.

ACR, albumin‐creatinine ratio; eGFR, estimated glomerular filtration rate; (THF+5αTHF)/THE, tetrahydrocortisol+5α‐tetrahydrocortisol/tetrahydrocortisone; 11β‐HSD, 11β‐hydroxysteroid dehydrogenase.

### 3.3 Diabetes mellitus

**Figure 3:**
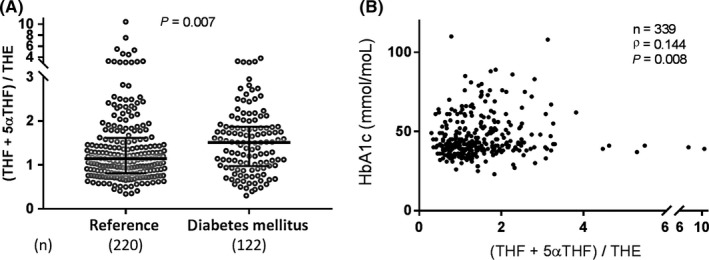
Association of urine (THF+5αTHF)/THE with diabetes mellitus and glycaemic control. A, Urinary (THF+5αTHF)/THE ratio was elevated in prevalent diabetes and (B) correlated positively with HbA1c, a measure of poor glycaemic control. (THF+5αTHF)/THE is displayed on a discontinuous scale to show outliers that were included in the analysis. Beams and whiskers indicate group medians and interquartile ranges, respectively; numbers in parentheses indicate cases per group. The Mann‐Whitney *U* test was used to compare groups; Spearman's test was used to assess correlations. (THF+5αTHF)/THE, tetrahydrocortisol+5α‐tetrahydrocortisol/tetrahydrocortisone

High 11β‐HSD1 activity leads to metabolic dysfunction and impaired insulin signalling, but this has not yet been tested in human CKD. Having identified that glucocorticoid activation by 11β‐HSD is elevated in CKD, we examined the association of urine (THF+5αTHF)/THE with prevalent diabetes. Participants with known diabetes mellitus had higher urine (THF+5αTHF)/THE (median [interquartile range (IQR)]: 1.51 [0.97‐1.87] vs 1.14 [0.81‐1.61], *P* = 0.007; Figure 3A). eGFR was not significantly different between those with or without diabetes (median [IQR]: 26.0 [16.5‐37.3] vs 29.1 [17.7‐47.5], *P* = 0.142), but CRP was higher in those with diabetes (median [IQR]: 3.40 [2.12‐9.51] vs 2.25 [1.08‐4.84], *P* < 0.001). We controlled for the potential confounding influence of CRP, eGFR and demographic factors by using multivariable logistic regression analysis (Table 3; urine (THF+5αTHF)/THE and eGFR were categorized for this analysis to comply with the requisite statistical assumptions as detailed in the methods section). The association of high urine (THF+5αTHF)/THE with diabetes was independent of these covariates. The association also persisted after the inclusion of body mass index and family history of diabetes as additional covariates. This supports a link between glucocorticoid activation by 11β‐HSD and diabetes mellitus in our CKD cohort.

**Table 3:** Adjusted odds ratio for the association of glucocorticoid activation by 11β‐HSD with prevalent diabetes mellitus

	n	Urine (THF+5αTHF)/THE						
		Low tertile	Middle tertile			High tertile		
			OR	95% CI	*P*	OR	95% CI	*P*
Unadjusted	342	Ref.	1.36	0.77‐2.42	0.289	2.57	1.47‐4.47	0.001
Model A	342	Ref.	1.30	0.70‐2.40	0.403	1.97	1.07‐3.62	0.029
Model B	331	Ref.	1.22	0.63‐2.35	0.556	2.09	1.09‐4.02	0.027

Binary logistic regression analysis for the association of urine (THF+5αTHF)/THE (stratified by tertiles) with prevalent diabetes. Model A: adjustment for age, sex, ethnicity, estimated glomerular filtration rate (stratified for ≤15, 15‐30, 30‐45 and >45 mL/min/1.73 m^2^) and C‐reactive protein (log_e_‐transformed). Model B: as in Model A plus adjustment for family history of diabetes and body mass index. The parameters for all covariates are reported in Table S4.

(THF+5αTHF)/THE, tetrahydrocortisol+5α‐tetrahydrocortisol/tetrahydrocortisone; 11β‐HSD, 11β‐hydroxysteroid dehydrogenase.

### 3.4 HbA1c and poor glycaemic control

To further corroborate the link between elevated GC activation by 11β‐HSD with impaired glucose metabolism in CKD, we assessed the relationship of GC activation by 11β‐HSD with HbA1c. HbA1c was positively correlated with urine (THF+5αTHF)/THE (*ρ* = 0.144, *P* = 0.008; Figure 3B). HbA1c also exhibited a negative association with eGFR (*ρ* = −0.127, *P* = 0.020) and a positive association with CRP (*ρ* = 0.337, *P* < 0.001) in our cohort. We controlled for these covariates and demographic factors in a multiple linear regression analysis. Age, South Asian ethnicity and CRP remained associated with HbA1c (*P* < 0.001, *P* < 0.001 and *P* = 0.001, respectively; Supporting Information Table S5), but urine (THF+5αTHF)/THE did not show an independent association in the full cohort (*P* = 0.659; Supporting Information Table S5). We considered that the association of urine (THF+5αTHF)/THE with HbA1c could be weakened by inclusion of the non‐diabetic population, as homoeostatic mechanisms that counter‐regulate increased insulin resistance are effective in this population and variability in HbA1c is low. Applying the same multiple linear regression model as before, we identified an independent association of urine (THF+5αTHF)/THE with HbA1c in the subgroup of patients with diabetes (*P* = 0.034; Supporting Information Table S5). Taken together, these results indicate an association of GC activation by 11β‐HSD and disturbances in glucose metabolism in the context of renal impairment.

### 3.5 Urinary cortisol‐cortisone ratio

A supplementary analysis of urinary cortisol‐cortisone ratio identified no significant association with eGFR, CRP, prevalent diabetes mellitus or HbA1c (Supporting Information Figure S3).

## 4 DISCUSSION

Previous studies in patients with CKD have suggested that increasing (THF+5αTHF)/THE ratios, favouring systemic cortisol activation, are determined by declining renal function and attenuated renal steroid clearance by 11β‐HSD2.^4^, ^5^ Our study confirmed that GC activation by 11β‐HSD is elevated in CKD, but identified inflammation as a crucial modifying factor in this setting. Furthermore, we identified an association between increasing (THF+5αTHF)/THE ratio with impaired glucose control in this CKD cohort.

Inflammation, measured as CRP, was the dominant explanatory variable for urine (THF+5αTHF)/THE in our data and significantly diminished the effect of eGFR as a covariate. The association between urine (THF+5αTHF)/THE and eGFR was not as strong as values previously reported in the literature.^4^, ^6^ Cohorts in previous studies were smaller, used sampling methodologies with greater potential for bias and included a majority of participants with eGFR >60 mL/min/1.73 m^2^. In contrast, the cohort in this study is more representative of a high‐risk CKD population.

Previous studies did not control for inflammation, which is concomitant with renal impairment. Inflammation is a potent inducer of 11β‐HSD1 expression and activity, with significant increases in cortisol activation by 11β‐HSD1 seen in several inflammatory diseases.^11^, ^12^, ^14^, ^34^, ^36^, ^37^ Whilst this study cannot accurately discriminate between the 11β‐HSD types 1 and 2 contributions to systemic GC metabolism, it is unlikely that our findings are solely attributable to loss of renal cortisol inactivation by 11β‐HSD2. The inflammatory cytokines TNFα and IL‐1β, which accumulate in uraemia, are potent inducers of 11β‐HSD1.^9^, ^11^ Furthermore, studies in human hepatocytes and animal models of uraemia report marked increases in 11β‐HSD1 activity.^7^, ^8^ Our results are therefore consistent with a growing body of evidence that increased inflammatory induction of 11β‐HSD1 contributes considerably to abnormal GC metabolism in patients with CKD.

In this study, we demonstrate that diabetes, and HbA1c among patients with diabetes, is associated with elevated urine (THF+5αTHF)/THE in CKD. The association of impaired glycaemic control with shifts in cortisol metabolism remained after adjustment for age, sex, ethnicity, renal function and inflammation. These results align with a large body of evidence that has established insulin resistance as a consequence of increased 11β‐HSD1 activity in animal models and human disease.^7^, ^23^, ^24^, ^26^, ^27^, ^28^, ^29^ The absence of a correlation between urine (THF+5αTHF)/THE and HbA1c in the non‐diabetic subgroup can be explained by a lack of change in HbA1c regardless of insulin resistance, as accompanying insulin secretion increases sufficiently to maintain glycaemic control.^18^ Our data therefore remain consistent with a role for 11β‐HSD1 in insulin resistance in human CKD.

Inflammation is a known driver for insulin resistance in CKD. This is evident by CRP showing a negative correlation with eGFR and an independent positive correlation with HbA1c in this study. Pro‐inflammatory cytokines TNFα and IL‐1 accumulate in CKD in serum, as well as in insulin target tissues, where they act on IRS‐1 and PKB/Akt to impede post‐receptor insulin signal transduction.^15^, ^16^ In the same tissues, in vitro experiments have demonstrated the capability of TNFα and IL‐1β to upregulate 11β‐HSD1, which in turn also causes insulin resistance through action on IRS‐1 and PKB/Akt.^11^, ^25^ Existing biochemical data therefore illustrate a coherent pathway through which inflammation‐mediated upregulation of 11β‐HSD1 leads to insulin resistance. Importantly, this opens 11β‐HSD1 inhibition as a therapeutic target in CKD to treat insulin resistance—and possibly other disturbances like dyslipidaemia—for which a proof of concept experiment in rodents has already been successful.^7^

Urinary cortisol‐cortisone ratio exhibited a moderate correlation with renal 11β‐HSD2 expression in a previous study by Quinkler et al^6^. An association between renal function and cortisol‐cortisone ratios in 24 hour urine samples has been described in some but not all studies.^4^, ^6^, ^40^ In this study, we did not identify any significant correlations between urinary cortisol‐cortisone ratios with eGFR, nor with CRP or Hb1Ac. These divergent results may reflect significant heterogeneity between study populations, or that spot urines lack sufficient sensitivity to detect these changes.

Several strengths and limitations are worth noting about this study. The observational design produced results that demonstrate associations between shifts in glucocorticoid metabolism, inflammation and impaired glucose metabolism, but cannot confirm causal relationships. By choosing this design, it was nevertheless possible to test predictions from pre‐clinical models, which link inflammation with raised 11β‐HSD1 activity and insulin resistance, in the clinical setting and thereby providing new evidence for the significance of this pathway in human disease. The study cohort was large and diverse, which permitted control for potential confounders, strengthens the validity of the results and enhances their relevance for people with CKD. Measuring urine metabolite ratios by liquid chromatography‐mass spectrometry as indicator of 11β‐HSD activity, whilst offering high convenience for a large study population, limits conclusions about 11β‐HSD type‐ or tissue‐specific alterations. Alterations in 11β‐HSD activity lead to much more pronounced shifts of cortisol‐cortisone equilibrium in local tissues than in circulation,^7^ meaning that correlations of inflammation and insulin resistance with local 11β‐HSD activities could be much stronger than what can be observed by measuring systemic metabolite ratios. Future research with methodologies that characterize 11β‐HSD type‐ and tissue‐specific alterations in humans with CKD will be valuable for building on our results. Finally, it would be relevant to investigate whether elevated GC activation by 11β‐HSD associates not only with impaired glycaemic control, but also with cardiovascular morbidity, progression of renal disease or mortality.

Examination of basal serum cortisol levels or total urinary GC excretion would add additional validation of our findings, helping delineate potential contributions of the hypothalamic‐pituitary‐adrenal (HPA) axis to our results. Without timed serum samples or urine samples from a fixed collection period for our cohort, we were unable to control directly for systemic cortisol levels or HPA axis activity. Nevertheless, previous studies validated the specificity of urine (THF+5αTHF)/THE for alterations in 11β‐HSD activity over changes in HPA axis activity and found no correlation between total GC metabolite excretion as a measure of HPA axis activity and renal function.^4^, ^6^, ^31^, ^32^ Neither do fluctuations in systemic cortisol interfere with urine (THF+5αTHF)/THE.^41^ Importantly, basal cortisol levels, as well as the fraction of protein bound cortisol (90%‐95%), do not change significantly with renal impairment.^42^, ^43^ We therefore argue that associations with urine (THF+5αTHF)/THE in our study reflect systemic 11β‐HSD regulation rather than systemic cortisol levels or HPA axis activity.

In conclusion, this study assessed systemic cortisol metabolism by 11β‐HSD enzymes in a large cohort of patients with pre‐dialysis chronic renal impairment. We identified inflammation as a major determinant of GC activation by 11β‐HSD and as a confounder in the correlation of GC activation with renal impairment, thereby expanding on knowledge from previous studies. Higher GC activation by 11β‐HSD was associated with disturbances in glucose homoeostasis, offering novel evidence for its clinical significance in patients with CKD. Our data agree with studies from preclinical and non‐renal settings that inflammation‐induced upregulation of 11β‐HSD1 can contribute to metabolic pathology. This study therefore adds credibility to the potential of 11β‐HSD1 inhibitors to reduce insulin resistance in populations with pro‐inflammatory conditions and improve their cardiovascular risk.

## DISCLOSURE

All authors declare that they have no relevant financial interests.

## AUTHORS' CONTRIBUTIONS

MSS, PGN, PC, RSH and LH contributed to research idea and study design. MSS, AET, AF, NAW and RSH involved in data acquisition. MSS, AET, AF, DC, PGN, CJF, WA, PC, RSH and LH performed data analysis/interpretation. MSS and PGN performed statistical analysis. AET, DC, PGN, CJF, WA, PC, RSH and LH involved in supervision or mentorship. Each author contributed important intellectual content during manuscript drafting or revision and accepts accountability for the overall work by ensuring that questions pertaining to the accuracy or integrity of any portion of the work are appropriately investigated and resolved.
