# Supplementary material for: Glucocorticoid activation by 11β‐hydroxysteroid dehydrogenase enzymes in relation to inflammation and glycaemic control in chronic kidney disease: A cross‐sectional study
Source: Clin Endocrinol (Oxf). 2018 Nov 15;90(1):241–9. doi: 10.1111/cen.13889 (PMC6334281; doi:10.1111/cen.13889)
Supplement: Supplementary file 1 [file CEN-90-241-s001.docx]

**Appendix S1: Supplementary Materials**

**Methods for measurement of urinary cortisol and cortisone**

Following THE, THF and 5αTHF quantification, the same sample was analysed on a separate method to measure cortisol and cortisone. Cortisol and cortisone were quantified relative to the internal standard cortisol-d4 (Sigma Aldrich, UK). The LC-MS/MS set up was identical to the initial THF/THE experiment except cortisol and cortisone were separated on a Waters HSS T3 1.2 x 50 mm column, with a gradient profile of 45% methanol (0.1% formic acid), hold 1min followed by a linear gradient to 98% methanol (0.1% formic acid) at 8.5 minutes. Cortisol and cortisone were identified by comparison to authentic reference standards, purchased from Sigma Aldrich, UK, with a matching retention time and identical mass transitions (MRMs) required for positive identification (supplementary Table S1 and supplementary Figure S2). Steroids were quantified relative to a calibration series ranging from 10-5000 ng/mL prepared in synthetic urine, including a blank. Validation of this method is described in the supplementary section (supplementary Table S2).


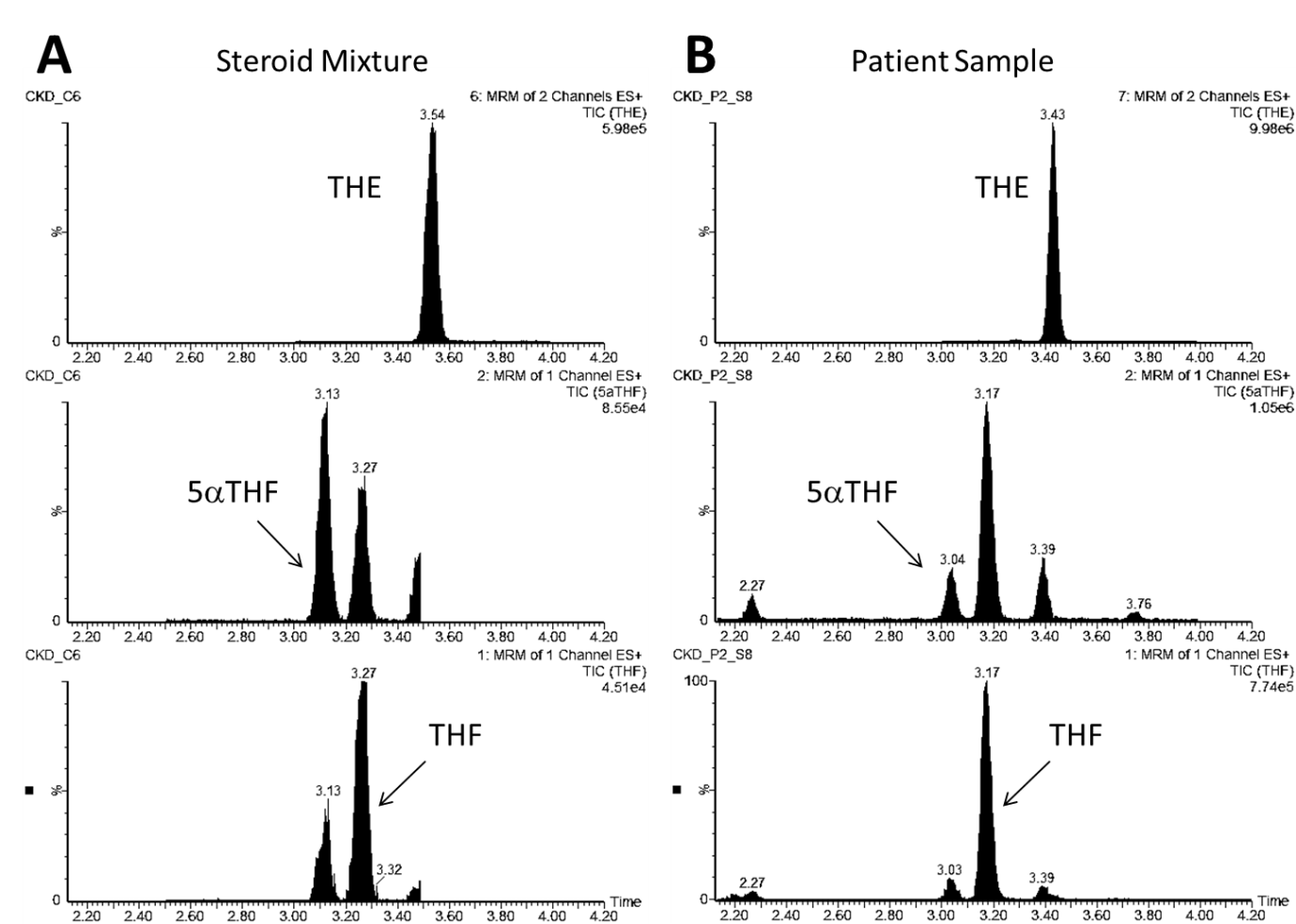


**a**

**b**

**Figure S1:** Chromatograms demonstrating separation of **(a)** authentic reference standards of THE, 5αTHF and THF and **(b)** detection of corresponding steroids in patient urine.


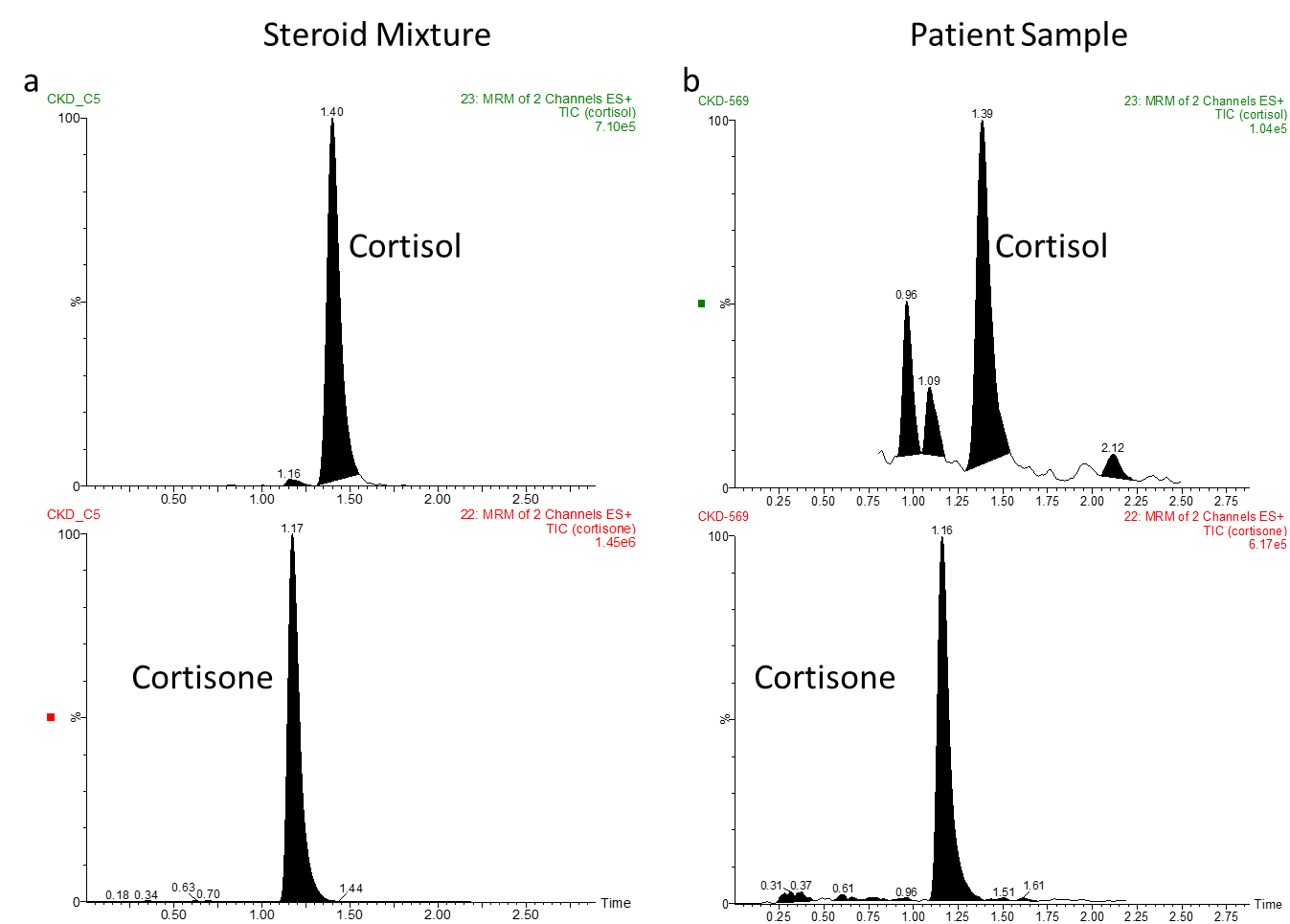


**Figure S2:** Chromatograms demonstrating separation of **(a)** authentic reference standards of cortisol and cortisone and **(b)** detection of corresponding steroids in patient urine.

**a**

**b**

**c**

**d**

**Figure S3. Supplementary analysis for urinary cortisol-cortisone ratio.** Urinary cortisol-cortisone ratio shows no significant associations with eGFR **(a)**, C-reactive protein **(b)**, diabetes mellitus **(c)** or HbA1c **(d)**. C-reactive protein is displayed on a log-scale. Beams and whiskers indicate group medians and interquartile range respectively. Spearman’s test was used to assess correlations with eGFR, C-reactive protein and HbA1c; the Mann-Whitney-U test was used to compare groups. eGFR: estimated glomerular filtration rate.

**Table S1.** Multiple-reaction monitoring mode mass transitions and mass spectrometer parameters for each steroid.

| Abb. | Name | MRM | Cone voltage (V) | Collision energy (eV) |
| --- | --- | --- | --- | --- |
| THF | Tetrahydrocortisol | 331.22 > 301.19 | 22 | 12 |
| 5αTHF | 5α-tetrahydrocortisol | 331.3 > 295.18 | 18 | 12 |
| *THF-d5* |  | *336.3 > 300.2* | *22* | *12* |
| THE | tetrahydrocortisone | 365.18 > 347.2 | 12 | 10 |
| *THE-d5* |  | *370.20 > 334.20* | *12* | *14* |
| F | Cortisol | 363.17 > 120.98 | 28 | 24 |
| *F-d4* | *Cortisol-d4* | *367.29 > 121.11* | *24* | *24* |
| E | Cortisone | 361.23 > 163.08 | 28 | 22 |

Abb: abbreviation; MRM: multiple-reaction monitoring mode

**Table S2.** Method validation for steroid quantification.

| Steroid | LOQ (μg/L) | Repro  RSD % | Accuracy  RSD % | | | Precision  RSD % | | | Matrix effects | Abs Recovery |
| --- | --- | --- | --- | --- | --- | --- | --- | --- | --- | --- |
|  |  |  | **L** | **M** | **H** | **L** | **M** | **H** | **%** | **%** |
| THE | 17 | 14.7 | 11 | 12 | 4.4 | 2.2 | 2.0 | 3.0 | 0.03 | 103 |
| 5αTHF | 15 | 6.7 | 8.8 | 15.3 | 5.0 | 8.9 | 7.6 | 5.3 | 2 | 98 |
| THF | 8 | 9.0 | 12.2 | 16.0 | 4.2 | 11.4 | 6.1 | 4.3 | 6 | 104 |
| Cortisol | 7 | 16 | 6.5 | 5.6 | 3.1 | 13.9 | 2.7 | 1.6 | -0.003 | 99 |
| Cortisone | 15 | 6 | 8.3 | 3.0 | 2.3 | 4.8 | 2.6 | 1.0 | -0.003 | 102 |

Limit of Quantitation was defined as the average signal-to-noise ratio of 10:1, observed in the patient samples. Accuracy and precision were determined for each steroid at 3 concentrations representing low, medium and high levels observed in a healthy adult population (THE, 250, 500, 5000ng/mL; THF and 5αTHF, 50, 500 and 5000 ng/mL; cortisol and cortisone, 50, 500 and 5000 ng/ml). Synthetic urine was spiked at these concentrations six times to determine accuracy and a single sample was injected 6 times to determine precision. Reproducibility was determined by extracting the same quality control urine sample 6 times over the experiment period (approx. 4 weeks) and calculating the variance between the concentrations, represented as the relative standard deviation (RSD%). Matrix Effects and absolute recovery were calculated by spiking urine pre and post extraction and comparing to a non-extracted control as described in *Taylor PJ. Matrix effects: the Achilles heel of quantitative high-performance liquid chromatography-electrospray-tandem mass spectrometry. Clin Biochem. 2005;38(4):328-334.* LOQ: limit of quantification; Repro: reproducibility; RSD: relative standard deviation; Abs Recovery: absolute recovery.

**Table S3.** Study cohort characteristics by renal diagnosis.

| **Variable** | **No renal disease** | **Polycystic renal disease** | **Glomerulo-nephritis** | **Interstitial nephritis** | **Not known & other** | **Ischaemic / hypertensive nephropathy** | **Obstructive / reflux disease** | **Diabetic nephropathy** | **Total** | **dc** |
| --- | --- | --- | --- | --- | --- | --- | --- | --- | --- | --- |
| 4 |  |  |  |  |  |  |  |  |  |  |
| n | 11 | 32 | 70 | 19 | 85 | 70 | 24 | 31 | 342 |  |
| 4 |  |  |  |  |  |  |  |  |  |  |
| Age (years) | 71 (69-76) | 51 (43-61) | 54 (43-63) | 62 (49-67) | 66 (56-78) | 73 (62-81) | 54 (32-73) | 63 (53-75) | 63 (50-75) | 100% |
| 4 |  |  |  |  |  |  |  |  |  |  |
| Female | 6 (55%) | 14 (44%) | 19 (27%) | 9 (47%) | 33 (39%) | 27 (39%) | 10 (42%) | 16 (52%) | 134 (39%) | 100% |
| 4 |  |  |  |  |  |  |  |  |  |  |
| White ethnicity | 11 (100%) | 26 (81%) | 47 (67%) | 15 (79%) | 61 (72%) | 45 (64%) | 17 (71%) | 20 (65%) | 242 (71%) | 100% |
| South Asian ethnicity | 0 (0%) | 2 (6%) | 14 (20%) | 1 (5%) | 14 (16%) | 19 (27%) | 6 (25%) | 6 (19%) | 62 (18%) |  |
| Black ethnicity | 0 (0%) | 4 (13%) | 7 (10%) | 3 (16%) | 10 (12%) | 6 (9%) | 1 (4%) | 4 (13%) | 35 (10%) |  |
| Other ethnicity | 0 (0%) | 0 (0%) | 2 (3%) | 0 (0%) | 0 (0%) | 0 (0%) | 0 (0%) | 1 (3%) | 3 (1%) |  |
| 4 |  |  |  |  |  |  |  |  |  |  |
| eGFR (ml/min/1.73m²) | 76 (70-81) | 37 (26-49) | 40 (24-65) | 22 (15-40) | 26 (17-36) | 24 (16-31) | 28 (16-43) | 22 (14-28) | 28 (18-43) | 100% |
| ACR (mg/mmol) | 1.0 (1.0-1.5) | 9.1 (4.9-20.4) | 94.0 (34.1-165.7) | 16.8 (4.4-59.3) | 52.4 (5.1-123.4) | 13.3 (1.0-73.0) | 85.6 (30.4-147.9) | 80.0 (9.0-239.0) | 35.5 (5.4-120.6) | 99.4% |
| Diabetes | 0 (0%) | 4 (13%) | 17 (24%) | 5 (26%) | 31 (36%) | 32 (46%) | 2 (8%) | 31 (100%) | 122 (36%) | 100% |
| HbA1c (mmol/mol) | 36 (35-37) | 39 (35-41) | 41 (37-45) | 40 (39-49) | 42 (39-49) | 45 (41-53) | 39 (35-42) | 57 (53-68) | 42 (38-50) | 99.1% |
| 4 |  |  |  |  |  |  |  |  |  |  |
| CRP (mcg/ml) | 0.8 (0.2-1.2) | 1.4 (0.7-1.9) | 2.0 (1.0-5.1) | 3.9 (2.0-7.6) | 3.4 (2.2-7.5) | 3.4 (1.8-8.7) | 3.6 (1.3-8.5) | 3.1 (2.0-6.9) | 2.7 (1.2-6.3) | 100% |
| 4 |  |  |  |  |  |  |  |  |  |  |
| (THF + 5αTHF) / THE | 0.75 (0.62-0.90) | 0.99 (0.73-1.26) | 1.19 (0.75-1.84) | 1.21 (0.86-1.83) | 1.28 (0.91-1.78) | 1.29 (0.91-2.06) | 1.29 (1.16-1.64) | 1.50 (1.09-1.89) | 1.23 (0.86-1.80) | 100% |

Data is shown as median (interquartile range) for continuous variables and as frequency (percentage) for categorical variables. dc: data completeness; eGFR: estimated glomerular filtration rate; ACR: urinary albumin-creatinine ratio; CRP: C-reactive protein.

**Table S4.** Odds ratios for the association of glucocorticoid activation by 11bHSD with prevalent diabetes mellitus, including effects of covariates

| **Model** | **Variable** | | **odds ratio** | **95% CI** | | **p** |
| --- | --- | --- | --- | --- | --- | --- |
|  |  |  |  | **lower** | **upper** |  |
| **unadjusted** | (THF+5αTHF)/THE tertile | low | reference |  |  |  |
| n=342 |  | middle | 1.36 | 0.77 | 2.42 | 0.289 |
|  |  | high | 2.57 | 1.47 | 4.47 | 0.001 |
| **Model A** | (THF+5αTHF)/THE tertile | low | reference |  |  |  |
| n=342 |  | middle | 1.30 | 0.70 | 2.40 | 0.403 |
|  |  | high | 1.97 | 1.07 | 3.62 | 0.029 |
|  | Age (years) | | 1.03 | 1.01 | 1.05 | <0.001 |
|  | Sex | male | reference |  |  |  |
|  |  | female | 1.05 | 0.64 | 1.73 | 0.849 |
|  | Ethnicity | white | reference |  |  |  |
|  |  | south asian | 2.48 | 1.33 | 4.65 | 0.004 |
|  |  | black | 1.46 | 0.65 | 3.29 | 0.357 |
|  |  | other | 10.26 | 0.83 | 127.20 | 0.070 |
|  | eGFR | >45 | reference |  |  |  |
|  |  | 30-45 | 1.50 | 0.71 | 3.19 | 0.288 |
|  |  | 15-30 | 0.97 | 0.46 | 2.03 | 0.935 |
|  |  | <15 | 0.97 | 0.43 | 2.20 | 0.941 |
|  | CRP [log_e_-transformed] | | 1.35 | 1.09 | 1.66 | 0.005 |
| **Model B** | (THF+5αTHF)/THE tertile | low | reference |  |  |  |
| N=331 |  | middle | 1.22 | 0.63 | 2.35 | 0.556 |
|  |  | high | 2.09 | 1.09 | 4.02 | 0.027 |
|  | Age (years) |  | 1.05 | 1.03 | 1.07 | <0.001 |
|  | sex | male | reference |  |  |  |
|  |  | female | 0.96 | 0.56 | 1.67 | 0.891 |
|  | Ethnicity | white | reference |  |  |  |
|  |  | south asian | 3.02 | 1.53 | 5.96 | 0.001 |
|  |  | black | 1.23 | 0.50 | 3.00 | 0.657 |
|  |  | other | 14.09 | 1.04 | 191.28 | 0.047 |
|  | eGFR | >45 | reference |  |  |  |
|  |  | 30-45 | 1.35 | 0.59 | 3.10 | 0.477 |
|  |  | 15-30 | 0.84 | 0.37 | 1.92 | 0.683 |
|  |  | <15 | 0.86 | 0.35 | 2.12 | 0.750 |
|  | CRP [log_e_-transformed] | | 1.14 | 0.90 | 1.43 | 0.281 |
|  | Family History of Diabetes | no | reference |  |  |  |
|  |  | yes | 1.48 | 0.85 | 2.60 | 0.168 |
|  | Body-mass index (kg/m²) | | 1.14 | 1.09 | 1.19 | <0.001 |

CI: confidence interval; eGFR: estimated glomerular filtration rate; CRP: C-reactive protein.

**Table S5.** Multivariable linear regression model for HbA1c including effects of covariates

| **Group** | **Variable** | **B** | **Std. Error** | **Beta** | **t** | **p** |
| --- | --- | --- | --- | --- | --- | --- |
| **full cohort** | Age (years) | 4.16E-03 | 8.30E-04 | 0.281 | 5.011 | <0.001 |
|  | Female | 2.00E-02 | 2.60E-02 | 0.040 | 0.769 | 0.442 |
| n=339 | South Asian ethnicity | 1.42E-01 | 3.38E-02 | 0.221 | 4.200 | <0.001 |
|  | Black ethnicity | -4.75E-03 | 4.24E-02 | -0.006 | -0.112 | 0.911 |
| R²=0.124 | Other ethnicity | 7.24E-02 | 1.34E-01 | 0.028 | 0.539 | 0.590 |
|  | eGFR (ml/min/1.73m²) | 3.83E-04 | 6.03E-04 | 0.035 | 0.635 | 0.526 |
|  | CRP (mcg/ml) | 3.35E-03 | 9.57E-04 | 0.186 | 3.498 | 0.001 |
|  | (THF+5αTHF)/THE | 5.91E-03 | 1.34E-02 | 0.023 | 0.442 | 0.659 |
| **non-diabetic** | Age (years) | 2.13E-03 | 4.90E-04 | 0.319 | 4.336 | <0.001 |
|  | Female | 8.42E-03 | 1.64E-02 | 0.035 | 0.512 | 0.609 |
| n=217 | South Asian ethnicity | 2.68E-02 | 2.41E-02 | 0.078 | 1.112 | 0.267 |
|  | Black ethnicity | 8.93E-03 | 2.71E-02 | 0.023 | 0.330 | 0.742 |
| R²=0.072 | Other ethnicity | 6.52E-02 | 1.16E-01 | 0.037 | 0.562 | 0.575 |
|  | eGFR (ml/min/1.73m²) | -2.10E-04 | 3.64E-04 | -0.041 | -0.578 | 0.564 |
|  | CRP (mcg/ml) | -2.57E-04 | 7.66E-04 | -0.024 | -0.336 | 0.737 |
|  | (THF+5αTHF)/THE | 8.35E-04 | 7.77E-03 | 0.008 | 0.107 | 0.915 |
| **diabetic** | Age (years) | 4.11E-04 | 1.73E-03 | 0.023 | 0.238 | 0.812 |
|  | Female | 7.16E-02 | 4.16E-02 | 0.154 | 1.722 | 0.088 |
| n=122 | South Asian ethnicity | 1.07E-01 | 4.66E-02 | 0.204 | 2.297 | 0.023 |
|  | Black ethnicity | -1.35E-01 | 6.53E-02 | -0.185 | -2.072 | 0.041 |
| R²=0.119 | Other ethnicity | -1.31E-01 | 1.56E-01 | -0.074 | -0.836 | 0.405 |
|  | eGFR (ml/min/1.73m²) | 1.23E-03 | 1.04E-03 | 0.118 | 1.182 | 0.240 |
|  | CRP (mcg/ml) | 3.63E-03 | 1.17E-03 | 0.276 | 3.116 | 0.002 |
|  | (THF+5αTHF)/THE | 5.94E-02 | 2.77E-02 | 0.188 | 2.140 | 0.034 |

The dependent variable HbA1c was log-transformed. eGFR: estimated glomerular filtration rate; CRP: C-reactive protein. Reference groups for respective categorical variables were male sex and white ethnicity.
